# Association Between Baseline Glucagon-like Peptide-1 Receptor Agonist Use and Newly Documented Depression Diagnosis in Adults with Type 2 Diabetes: A Multi-State Medicaid Cohort Study

**DOI:** 10.3390/healthcare14182971

**Published:** 2026-09-11

**Authors:** Michelle Ndiulor, Hao Wang, Rolake Neba, Rafia Rasu, Bo Zhou, Usha Sambamoorthi

**Affiliations:** 1 Department of Pharmacotherapy, College of Pharmacy, UNT Health, 3500 Camp Bowie Blvd, Fort Worth, TX 76107, USA; michelle.ndiulor@unthsc.edu (M.N.); rafia.rasu@unthsc.edu (R.R.); bo.zhou@unthsc.edu (B.Z.); usha.sambamoorthi@temple.edu (U.S.); 2 Department of Emergency Medicine, JPS Health Network, 1500 S. Main St., Fort Worth, TX 76104, USA; hwang@ies.healthcare; 3 Department of Pharmacy Practice, Temple University School of Pharmacy, Philadelphia, PA 19122, USA

**Keywords:** glucagon-like peptide-1 receptor agonists, type 2 diabetes mellitus, newly documented depression

## Abstract

**Highlights:**

**What are the main findings?**

- In this large longitudinal cohort of Medicaid beneficiaries with T2DM, baseline GLP-1 RA use was associated with an increased likelihood of newly documented depression diagnosis during the subsequent 12-month follow-up.
- The observed association was modest and should not be interpreted as evidence of a causal relationship between GLP-1 RA use and depression.

**What are the implications of the main findings**

- These findings contribute to the limited evidence regarding GLP-1 RA use and depression among working-age Medicaid beneficiaries with T2DM.
- Further prospective studies with detailed medication exposure, standardized psychiatric assessment, and long-term follow-up are warranted to clarify the neuropsychiatric safety profile of GLP-1 RA.

**Abstract:**

**Background/Objectives**: Glucagon-like peptide-1 receptor agonists (GLP-1 RAs) are increasingly prescribed for type 2 diabetes mellitus (T2DM). However, its mental health safety profile in routine care, particularly among Medicaid beneficiaries, remains uncertain. We aim to examine the association between glucagon-like peptide-1 receptor agonist (GLP-1 RA) use and newly documented depression diagnosis among adults with type 2 diabetes mellitus (T2DM) enrolled in Medicaid. **Methods**: We conducted a retrospective longitudinal cohort study using the 2022–2023 Merative™ MarketScan^®^ Multi-State Medicaid Database. Adults aged 18–64 years with T2DM, continuous Medicaid enrollment, and no documented depression during the 2022 baseline period were included. Baseline GLP-1 RA use was assessed in 2022, and newly diagnosed depression was identified in 2023. Associations were estimated using multivariable logistic regression, inverse probability weighting (IPW), and a doubly robust estimator. **Results**: Among 74,760 eligible adults, 14,953 (20.0%) were GLP-1 RA users. Incident depression occurred in 12.9% of users and 11.1% of non-users (absolute difference, 1.8 percentage points; *p* < 0.001). GLP-1 RA use was associated with higher odds of newly documented depression diagnosis in multivariable analysis (adjusted OR 1.10, 95% CI 1.04–1.16), IPW analysis (OR 1.09, 95% CI 1.03–1.15), and doubly robust analysis (OR 1.09, 95% CI 1.03–1.15). **Conclusions**: Baseline GLP-1 RA use was associated with modestly higher odds of a newly documented depression diagnosis during the subsequent year. Prospective studies are needed to determine whether this association is causal.

## 1. Introduction

Glucagon-like peptide-1 receptor agonists (GLP-1 RAs) have become increasingly important in the management of type 2 diabetes mellitus (T2DM). These medications augment glucose-dependent insulin secretion, suppress glucagon release, delay gastric emptying, and promote satiety, thereby improving glycemic control and achieving clinically meaningful weight loss [1,2,3]. Selected GLP-1 RAs also reduce major cardiovascular events and improve renal outcomes in patients at elevated cardiometabolic risk, further expanding their role in contemporary diabetes care [4,5]. As their use increases, understanding outcomes beyond glycemic and cardiovascular benefits, including potential neuropsychiatric effects, has become clinically relevant.

The relationship between GLP-1 signaling and mood is biologically plausible, although its direction remains uncertain. GLP-1 receptors are expressed in several regions of the central nervous system involved in appetite, reward, motivation, stress responses, and emotional regulation [6]. Experimental studies suggest that GLP-1 signaling can influence dopaminergic reward pathways, neuroinflammation, oxidative stress, and hippocampal neuroplasticity [6,7]. These actions could theoretically improve depressive symptoms by improving metabolic control and attenuating inflammatory processes. Conversely, alterations in reward processing, appetite, energy intake, or treatment-related symptoms could potentially affect mood in susceptible individuals [8]. Therefore, the neuropsychiatric effects of GLP-1 RAs may be heterogeneous and cannot be inferred solely from their metabolic benefits.

Clinical evidence regarding psychiatric outcomes associated with GLP-1 RAs has been inconsistent. A recent 2025, 2026 meta-analysis of randomized placebo-controlled trials, including approximately 46,000 GLP-1 RA–treated participants in the analysis of serious psychiatric adverse events, found no significant difference between GLP-1 RAs and placebo in either serious or nonserious psychiatric adverse events [9]. However, psychiatric outcomes were generally secondary safety endpoints, event numbers were low, and patients with substantial psychiatric histories were frequently underrepresented or excluded from pivotal trials. Furthermore, These trials generally evaluated broader psychiatric adverse-event outcomes rather than depression as a prespecified primary outcome. In contrast, a propensity score–matched observational study of adults with obesity compared individuals receiving liraglutide or semaglutide with individuals not receiving GLP-1 RA therapy and reported an association between GLP-1 RA treatment and major depressive disorder [10]. However, the non-active comparator design and the magnitude of the reported association raise concerns regarding residual confounding, differences in healthcare utilization, and confounding by indication. Pharmacovigilance studies have also identified reports of psychiatric adverse events among GLP-1 RA users. For example, an analysis of the US Food and Drug Administration Adverse Event Reporting System identified 8240 psychiatric adverse-event reports among 181,238 GLP-1 RA–associated reports; however, spontaneous-reporting databases cannot estimate incidence or establish causality because of selective reporting, incomplete clinical information, and the absence of an appropriate untreated comparison group [11].

Evaluating this association is particularly challenging because both T2DM and obesity are independently associated with depression [12]. In a US population-based analysis, the prevalence of depression among adults with diabetes was 29.2%, compared with 17.9% among adults without diabetes [13]. Depression may also influence medication adherence, healthcare utilization, glycemic control, body weight, and treatment selection [14,15]. Consequently, an apparent association between GLP-1 RA use and depression could reflect differences in underlying disease burden, obesity, psychosocial circumstances, access to care, or clinical surveillance.

Medicaid beneficiaries are an important population in which to examine this question. Individuals enrolled in Medicaid may experience a disproportionate burden of chronic illness, behavioral-health conditions, social adversity, and barriers to continuity of care [16,17]. At the same time, Medicaid coverage policies and access to newer diabetes medications vary across plans and jurisdictions. These factors may affect both the selection of patients for GLP-1 RA treatment and the detection of depression in routine practice. Nevertheless, Medicaid beneficiaries have been underrepresented in clinical trials and many observational evaluations of GLP-1 RA-related psychiatric outcomes, leaving limited evidence specific to working-age Medicaid beneficiaries with T2DM.

Therefore, we conducted a longitudinal cohort study using the Merative MarketScan Multi-State Medicaid Database to examine whether GLP-1 RA use during a 12-month baseline period was associated with newly diagnosed depression during the subsequent 12 months among adults with T2DM and no documented baseline depression. We used conventional multivariable adjustment, inverse probability weighting, and a doubly robust estimator to evaluate the consistency of the association after accounting for measured demographic, psychosocial, insurance, and clinical characteristics. We hypothesized that GLP-1 RA exposure would be associated with a difference in the likelihood of receiving a new depression diagnosis during follow-up; given the conflicting prior evidence, the direction of this association was not prespecified.

## 2. Materials and Methods

### 2.1. Study Design and Data Source

We conducted a retrospective, claims-based, longitudinal cohort study using the 2022–2023 Merative™ MarketScan^®^ Multi-State Medicaid Database (Merative, Ann Arbor, MI, USA). This administrative claim database contains de-identified enrollment information and medical and pharmacy claims from Medicaid beneficiaries enrolled in both fee-for-service and managed care plans across multiple U.S. states. The database includes patient demographics, inpatient and outpatient diagnoses and procedures, outpatient prescription dispensing records, and enrollment information, allowing longitudinal follow-up of individual beneficiaries across healthcare settings.

To establish the temporal relationship between medication exposure and study outcome, we divided the study period into two consecutive windows. 1 January 2022, served as the study time origin, with eligible beneficiaries entering the baseline assessment period on that date. The baseline (exposure) period extended from 1 January through 31 December 2022, during which GLP-1 RA exposure and baseline covariates were ascertained. The follow-up (outcome) period extended from 1 January through 31 December 2023, during which newly diagnosed depression was identified. We required continuous enrollment in baseline and follow-up year; short enrollment gaps were not permitted. Continuous Medicaid enrollment throughout both periods was required to ensure complete ascertainment of medication exposure, baseline characteristics, and study outcomes. The study was approved by the University of North Texas Health Science Center Regional Institutional Review Board with a waiver of informed consent because only de-identified administrative claims data were analyzed. The study was conducted in accordance with the Declaration of Helsinki. The study timeline, including cohort entry, baseline exposure and covariate assessment, and follow-up outcome ascertainment, is provided in the Supplementary Materials as Figure S2.

### 2.2. Study Cohort

Eligible participants were adults aged 18–64 years with T2DM who maintained continuous Medicaid enrollment with prescription drug coverage throughout the 2022 baseline and 2023 follow-up periods. T2DM was identified using a validated administrative claims algorithm based on ICD-10-CM diagnosis codes recorded during the baseline period. We required one inpatient claim, or two outpatient claims with 30 days apart, to validate T2DM diagnosis. To evaluate newly documented depression diagnosis, individuals with any diagnosis of depressive disorders during the baseline period were excluded. We also excluded beneficiaries who lacked continuous enrollment and prescription drug coverage during the 24-month study period, were not observed throughout the entire follow-up period due to death or disenrollment, or failed to meet the study eligibility criteria. The final study cohort, therefore, consisted of adults aged 18–64 years with T2DM who were continuously enrolled during 2022–2023, had no documented baseline depression, and had continuous insurance coverage in follow-up for claim-based outcome ascertainment. Eligible beneficiaries were continuously enrolled in Medicaid with prescription drug coverage throughout the 2022 baseline and 2023 follow-up periods.

### 2.3. Exposure to GLP-1 Receptor Agonists

The primary exposure was GLP-1 RA therapy during the baseline period. The study consisted of a 12-month baseline period from 1 January through 31 December 2022, during which GLP-1 RA exposure and baseline covariates were assessed, followed by a 12-month outcome period from 1 January through 31 December 2023, during which newly documented depression diagnoses were identified. Patients were classified as GLP-1 RA users if outpatient pharmacy claims demonstrated any dispensing of a GLP-1 RA medication during the baseline period, including a single dispensing or multiple/combination dispensing; repeated dispensing was not required for classification. Individuals who did not receive any GLP-1 RA dispensing during the baseline period were classified as non-users. GLP-1 RA agents represented in the study cohort included dulaglutide, exenatide, liraglutide, semaglutide, and tirzepatide. For the primary analysis, these agents were evaluated collectively as a medication class rather than as individual agents.

Exposure status was determined exclusively during the baseline period and remained fixed throughout the follow-up using a landmark study design. Consequently, GLP-1 RA use initiated after 1 January 2023 was not used to redefine exposure status. Likewise, changes in medication use during follow-up, including treatment discontinuation or initiation among baseline non-users, were not modeled. Among the 59,807 participants classified as GLP-1 RA non-users during the baseline period, 7338 (12.27%) had evidence of GLP-1 RA use during the 2023 follow-up period but remained classified as baseline non-users in the primary analysis. This design was selected to provide clear temporal separation between medication ascertainment and outcome assessment and to avoid overlapping exposure and outcome classification.

### 2.4. Outcome—New Documented Diagnosed Depression

The primary outcome was newly documented diagnosed depression during the follow-up period (1 January through 31 December 2023). Newly documented diagnosed depression was identified using ICD-10-CM codes recorded in inpatient or outpatient medical claims. A single qualifying diagnosis at any time during the follow-up year (2023) was sufficient to identify newly diagnosed depression. The use of a single recorded diagnosis to identify depression has precedent in administrative claims research, including studies using Medicare and other large administrative databases [18,19,20]. The complete ICD-10-CM code list used to identify newly documented depression diagnoses is provided in Supplementary Table S1.

### 2.5. Covariates

Baseline covariates included demographic characteristics (age, sex, race and ethnicity), insurance characteristics (Medicaid plan type), obesity, selected substance use disorders (alcohol use disorder, opioid use disorder, and tobacco use), indicators of social determinants of health derived from ICD-10-CM Z codes, and overall medical comorbidity measured by the number of chronic conditions. These variables were selected a priori because they may influence both GLP-1 RA treatment selection and the subsequent risk of depression.

### 2.6. Statistical Analysis

Baseline characteristics were summarized using frequencies and percentages for categorical variables and means with standard deviations (SDs) for continuous variables. Medians and interquartile ranges were reported for continuous variables if distributions are skewed. Group comparisons were performed using chi-square tests for categorical variables and *t*-tests for continuous variables, as appropriate. Diagnosis and comorbidity indicators were coded as absent when no qualifying diagnosis code was identified in the claims during the specified assessment period. Missing data were limited to race/ethnicity and mental health coverage, which were retained as separate unknown/missing categories; no imputation was performed.

The primary analysis evaluated the association between baseline GLP-1 RA use and newly documented diagnosed depression during the follow-up using multivariable logistic regression with adjustment for prespecified demographic, clinical and psychosocial variables. Because treatment assignment was non-randomized, we performed two additional analyses to evaluate the robustness of the findings. Propensity scores for baseline GLP-1 RA use were estimated using logistic regression based on prespecified baseline demographic, insurance, social, behavioral, and clinical characteristics. Raw inverse probability weights were for GLP-1 RA users and non-users. The inverse probability weights (IPWs) used in the analyses were normalized separately within the exposure groups by dividing each raw inverse probability weight by the mean raw weight within the corresponding exposure group. Therefore, the mean normalized weight was approximately 1.0 in each exposure group. No truncation or trimming of the weights was performed.

The normalized weights ranged from 0.43 to 4.85. Propensity scores ranged from 0.04 to 0.46 among GLP-1 RA users and from 0.03 to 0.49 among non-users. The distributions showed substantial overlap between the two exposure groups, with the propensity score range among GLP-1 RA users contained within the range observed among non-users. The effective sample size after weighting was 72,169 compared with an unweighted sample size of 74,760. Group-specific effective sample sizes were 13,049 among GLP-1 RA users and 59,310 among non-users. Covariate balance before and after weighting was evaluated using absolute standardized mean differences, with an absolute SMD < 0.10 considered indicative of adequate balance. All measured baseline covariates met this criterion after weighting.

Weighted outcome models were estimated using SAS PROC SURVEYLOGISTIC (SAS Institute, Cary, NC, USA). with the normalized inverse probability weights. Variances and standard errors were estimated using the Taylor-series linearization approach with 95% Wald confidence intervals. In addition to the IPW model, an IPW-weighted multivariable logistic regression was fit, which included GLP-1 RA exposure and baseline demographic, insurance, social, and clinical covariates.

We compared estimates across these complementary analytical approaches to assess the consistency of the association after adjustment for measured confounding. Similar estimates across models were interpreted as evidence of consistency across analytical approaches rather than as confirmation that residual or unmeasured confounding had been eliminated. Odds ratios (ORs) and 95% confidence intervals (CIs) were reported. All statistical tests were two-sided, and *p*-values < 0.05 were considered statistically significant. Statistical analyses were performed using SAS version 9.4 (SAS Institute, Cary, NC, USA).

## 3. Results

### 3.1. Study Cohort Characteristics

A total of 74,760 adults aged 18–64 years with T2DM met the study eligibility criteria and were included in the final analysis. Among them, 14,953 (20.0%) were classified as GLP-1 RA users during the baseline period, whereas 59,807 (80.0%) had no documented GLP-1 RA use and served as the comparison group. Baseline characteristics before and after the IPW are summarized in Table 1. Before weighting, GLP-1 RA users were more likely to be female, middle-aged (35–54 years), non-Hispanic White, obese, and enrolled in capitated Medicaid plans than non-users. After the IPW, all measured baseline covariates demonstrated adequate balance, with absolute standardized mean differences below 0.10, indicating successful reduction in measured baseline differences between exposure groups.

### 3.2. Newly Documented Depression Diagnosis

During the 12-month follow-up period, 8561 participants (11.5%) received a new documentation diagnosis of depression. Newly documented depression diagnosis was identified in 1936 of 14,953 GLP-1 RA users (12.9%) and 6625 of 59,807 non-users (11.1%) (12.9% vs. 11.1%; *p* < 0.001), corresponding to an unadjusted absolute difference of 1.8 percentage points. Although the absolute risk difference was 1.8 percentage points, the association remained statistically significant after adjustment for measured confounders using three complementary analytical approaches. Detailed baseline characteristics according to depression status are shown in Table 2.

### 3.3. Association Between GLP-1 RA Use and Newly Documented Depression Diagnosis

Table 3 summarizes the associations between baseline GLP-1 RA use and newly documented diagnosed depression estimated using three complementary analytical approaches. In the multivariable logistic regression model adjusted for demographic characteristics, psychosocial factors, and clinical comorbidities, GLP-1 RA use was associated with higher odds of newly documented depression diagnosis (adjusted OR, 1.10; 95% CI, 1.04–1.16). After inverse probability weighting to improve baseline covariate balance, the association remained similar (marginal OR, 1.09; 95% CI, 1.03–1.15). The doubly robust estimator produced nearly identical results (OR, 1.09; 95% CI, 1.03–1.15).

Across all three analytical approaches, the estimated association between GLP-1 RA use and newly documented depression diagnosis was similar across the multivariable, IPW, and doubly robust analyses.

To provide a more directly interpretable relative measure, we conducted a modified Poisson regression with a log link and robust standard errors, using the same IPWs and covariates included in the fully adjusted analysis. GLP-1 RA use was associated with an 8% higher adjusted risk of newly documented depression compared with non-use (adjusted RR, 1.08; 95% CI, 1.02–1.13; *p* = 0.0041). The adjusted absolute risk was 12.11% among GLP-1 RA users and 11.25% among non-users, corresponding to an adjusted risk difference of 0.85 percentage points. Thus, although the relative risk was modestly elevated, the absolute difference in risk was less than 1 percentage point. This estimate was consistent with the magnitude and direction of the association observed in the logistic regression analyses.

## 4. Discussion

In this large longitudinal cohort of Medicaid beneficiaries with T2DM, baseline GLP-1 RA use was associated with an increased likelihood of newly documented depression diagnosis during the subsequent 12-month follow-up. This association was consistent across conventional multivariable logistic regression, inverse probability weighting, and a doubly robust estimator, showing consistency across complementary analyses addressing measured confounding. Although the unadjusted absolute risk difference was 1.8 percentage points, the adjusted association was small (aOR approximately 1.09–1.10). Given the large sample size, statistical significance should not be interpreted as indicating a large clinical effect. These findings should be interpreted in the context of the observational study design, which precludes causal inference.

Our findings should be interpreted within the context of an evolving, sometimes conflicting, literature. Randomized clinical trials and recent 2025, 2026 meta-analyses have generally not demonstrated an increased risk of psychiatric adverse events among individuals receiving GLP-1 RAs, although psychiatric outcomes were rarely primary endpoints and patients with significant psychiatric illness were frequently excluded [9,21]. One study using an active-comparator design did not find evidence of an increased risk of suicidal behavior among GLP-1 RA users compared with users of other glucose-lowering therapies [22]. Conversely, several pharmacovigilance studies and observational investigations have reported increased reporting of depression or other psychiatric adverse events among GLP-1 RA users [23,24]. These findings should be interpreted cautiously, as pharmacovigilance studies can identify potential safety signals but cannot estimate comparative incidence or establish causal associations and are susceptible to reporting and other biases. Regulatory evidence has also been reassuring regarding suicidality. In a comprehensive review, the U.S. Food and Drug Administration did not identify an increased risk of suicidal ideation or behavior with GLP-1 RA use; however, these findings primarily address suicidality and do not directly resolve the association between GLP-1 RA use and depression [25]. Differences in study populations, comparator selection, outcome definitions, exposure assessment, duration of follow-up, healthcare utilization, outcome surveillance, and susceptibility to residual confounding may contribute to these inconsistent findings. Our study extends this literature by examining a large Medicaid population using a longitudinal design in which baseline medication exposure was assessed before follow-up outcome ascertainment. However, this temporal ordering does not establish causality or eliminate the potential for reverse causation, residual confounding, or other sources of bias.

Several biological mechanisms have been proposed to explain how GLP-1 signaling could influence mood, although current evidence remains inconclusive. GLP-1 receptors are widely distributed throughout the central nervous system and participate in neural pathways involved in reward processing, stress responses, appetite regulation, and neuroinflammation [26,27]. These mechanisms suggest that GLP-1 signaling may influence emotional regulation in susceptible individuals. At the same time, improved glycemic control, weight reduction, and reduced systemic inflammation associated with GLP-1 RA therapy could, in theory, improve psychological well-being [28]. Therefore, the association observed in this study should not be interpreted as evidence that GLP-1 RAs directly cause depression, but rather as an association warranting further investigation. Clinicians should continue to consider patients’ overall mental health and clinical context when initiating and monitoring therapy, while recognizing that the present findings alone do not demonstrate an adverse psychiatric effect of GLP-1 RAs.

The Medicaid context is also important when interpreting these findings. Within our cohort, newly documented depression diagnoses were more common among beneficiaries with recorded social needs (17.6%) than among those without recorded social needs (11.3%). Higher percentages were also observed among beneficiaries with recorded alcohol use (18.3%), opioid use disorder (18.8%), and tobacco use (17.9%). These descriptive differences highlight the substantial social and behavioral health complexity within this Medicaid population and identify factors that may be related to both treatment patterns and the documentation of depression. However, these unadjusted percentages should not be interpreted as evidence that these characteristics caused depression or explain the observed association between GLP-1 RA use and newly documented depression.

Our study has several strengths. First, we analyzed a large, multi-state Medicaid cohort representing a population that is often underrepresented in randomized clinical trials. Second, the longitudinal cohort design established temporal separation between baseline medication exposure and subsequent depression diagnoses. Third, the consistency of the findings across multivariable regression, inverse probability weighting, and doubly robust estimation demonstrates consistency across complementary approaches used to address measured baseline characteristic difference.

Several limitations should also be considered. First, this was an observational study using administrative claims data; therefore, causal relationships cannot be established, and residual confounding from unmeasured factors remains possible. Although IPW achieved balance across measured covariates, factors such as HbA1c, duration and severity of diabetes, prior treatment response, and subclinical or undiagnosed mental health symptoms were not fully captured and may have influenced both GLP-1 RA treatment selection and subsequent depression diagnosis. Second, depression was identified using ICD-10-CM diagnosis codes, claims data capture clinically recognized and coded depression, and may not identify undiagnosed, unrecognized, or uncoded depression, which may potentially result in outcome misclassification. Differences in healthcare utilization may also have influenced opportunities for depression recognition and diagnosis coding, potentially introducing detection bias. Third, exposure classification was based on any GLP-1 RA dispensing during the baseline period and does not establish medication adherence, treatment persistence, or continuous exposure. Changes in medication use during follow-up, including treatment discontinuation or initiation after baseline, were not modeled. Consequently, fixed baseline exposure classification may have resulted in exposure misclassification when treatment changed during follow-up, potentially attenuating or otherwise distorting the observed association. Fourth, although individual GLP-1 RA agents were identifiable in the claims, the primary analysis evaluated GLP-1 RAs as a medication class and did not estimate agent-specific associations with newly documented depression. Potential differences in neuropsychiatric outcomes across individual agents therefore could not be assessed and warrant further investigation. Fifth, detailed information on concurrent antidiabetic medications was not incorporated into the present analysis. GLP-1 RA users may differ from non-users in their use of insulin and other glucose-lowering therapies and in overall treatment intensity. Therefore, residual confounding related to diabetes severity and treatment intensity cannot be ruled out. Sixth, the requirement for continuous Medicaid enrollment may have introduced selection bias and may limit the generalizability of the findings to other healthcare populations. Individuals who maintained continuous enrollment may differ systematically from those who did not, which may limit the generalizability of the findings. In addition, because the study population was restricted to Medicaid beneficiaries aged 18–64 years, the findings may have limited generalizability to adults aged 65 years or older, Medicare beneficiaries, commercially insured populations, and uninsured individuals. Potential heterogeneity by age, sex, obesity status, and individual GLP-1 RA agent was not evaluated and should be examined in future studies with sufficiently detailed medication exposure data.

Lastly, residual confounding and confounding by indication remain important limitations of this observational study. Although IPW achieved good balance across the measured baseline characteristics, including demographic, insurance, social, behavioral, and clinical factors, weighting can account only for variables that were measured and included in the propensity score model. Factors that may influence both the selection of GLP-1 RA therapy and depression risk, including diabetes severity, HbA1c, duration of diabetes, stage of diabetes treatment, cardiovascular risk, treatment intensity, and healthcare engagement, were either unavailable or incompletely captured in the claims data and therefore could not be fully accounted for. In addition, the non-user comparison group was heterogeneous and may have included individuals at different stages of diabetes treatment and with different indications or contraindications for GLP-1 RA therapy. Consequently, differences between GLP-1 RA users and non-users may persist despite the observed post-weighting covariate balance, and the reported association should not be interpreted as establishing a causal effect of GLP-1 RA use on newly documented depression. The study also classified GLP-1 RA exposure based on any use during the baseline period rather than employing a new-user, active-comparator design. Consequently, GLP-1 RA users may have included both established and more recent users, while the non-user group represented a heterogeneous comparison population that may have differed in treatment history and stage of diabetes management. This design may contribute to prevalent-user bias and residual confounding that cannot be fully addressed through adjustment for measured covariates. Future studies should consider new-user, active-comparator designs comparing patients initiating GLP-1 RAs with patients initiating alternative glucose-lowering therapies used at a similar stage of treatment, such as sodium-glucose cotransporter-2 (SGLT2) inhibitors.

## 5. Conclusions

Among adults with T2DM enrolled in Medicaid, baseline GLP-1 RA use was associated with an increased likelihood of newly documented depression diagnosis during the subsequent year. Although the association was consistent across multiple analytical approaches, the observational nature of the study precludes causal inference. Further prospective studies with detailed medication exposure, standardized psychiatric assessment, and long-term follow-up are warranted to clarify the neuropsychiatric safety profile of GLP-1 RA.

## Figures and Tables

**Table 1:** Baseline Characteristics Before and After Inverse Probability Weighting.

	Before IPW			After IPW		
	GLP1-RA User	Non-User	Abs. SMD	GLP1-RA User	Non-User	Abs. SMD
	%	%		Wt. %	Wt. %	
Sex			0.1658			0.0013
Female	63.5	55.3		56.9	57.0	
Male	36.5	44.7		43.1	43.0	
Age in Years						
18–34 Years	8.8	10.6	0.0579	10.2	10.2	0.0021
35–44 Years	19.9	17.1	0.0740	17.7	17.6	0.0010
45–54 Years	31.9	27.2	0.1037	28.1	28.1	0.0010
55–64 Years	39.3	45.2	0.1193	44.1	44.1	0.0015
Race and Ethnicity						
NHW	46.1	37.9	0.1660	39.6	39.6	0.0012
NHB	20.7	25.1	0.1045	24.0	24.2	0.0037
Hispanic	14.1	11.7	0.0716	12.4	12.2	0.0072
Other Race	4.8	5.8	0.0447	5.7	5.6	0.0016
Capitation			0.1164			0.0010
Capitated	32.1	26.8		28.0	27.9	
FFS	67.9	73.2		72.0	72.1	
SDOH			0.0391			0.0034
Yes, problems	2.1	2.7		2.5	2.6	
No, problems	97.9	97.3		97.5	97.4	
Plan: Comprehensive			0.0334			0.0014
Comprehensive	68.0	69.6		69.2	69.3	
No Comp	32.0	30.4		30.8	30.7	
Plan: HMO			0.0964			0.0009
HMO	30.1	25.8		26.7	26.6	
No HMO	69.9	74.2		73.3	73.4	
Plan: PCCM			0.0918			0.0005
PCCM	7.7	5.5		5.9	5.9	
No PCCM	92.3	94.5		94.1	94.1	
Obesity			0.2715			0.0074
Obesity	62.2	48.8		51.9	51.5	
No, obesity	37.8	51.2		48.1	48.5	
Alcohol Use			0.1374			0.0025
Alcohol user	1.9	4.3		3.9	3.9	
Nonuser	98.1	95.7		96.1	96.1	
OUD			0.0476			0.0048
Yes, OUD	5.3	6.4		6.1	6.2	
No OUD	94.7	93.6		93.9	93.8	
Tobacco Use			0.0197			0.0022
Yes	0.7	0.9		0.8	0.8	
No	99.3	99.1		99.2	99.2	
# of Chronic Conditions	Mean (SD)	Mean (SD)		Mean (SD)	Mean (SD)	
	2.72 (1.64)	2.61 (1.68)	0.0665	2.64 (1.62)	2.63 (1.69)	0.0051
	Median (IQR)	Median (IQR)		Median (IQR)	Median (IQR)	
	3 (2)	2 (3)		2 (2)	2 (3)	

Notes: Based on 74,760 adults aged 18–64 years, continuously enrolled in Medicaid with prescription drug coverage during 2022 and 2023, with Type 2 Diabetes Mellitus. Percentages may not sum to 100 because missing values for race and ethnicity are not shown in the table. SMDs quantify the difference in baseline covariate balance between GLP-1 RA users and non-users. An SMD of >0.10 is generally considered to indicate imbalance. SMDs were independent of sample size and were computed before and after IPW application. Abbreviations: SMD: Absolute standardized mean difference; Comp: Comprehensive; FFS: Fee-for-service; GLP1-RA: Glucagon-like Peptide-1 Receptor Agonists; HMO: Health maintenance organization; NHB: Non-Hispanic Black; NHW: Non-Hispanic White; OUD: Opioid use disorder; PCCM: Primary care case management; SD: Standard deviation; SDOH: Social determinants of health; Wt: Weighted.

**Table 2:** Description of Study Cohort by Newly Documented Depression Diagnosis Adults (age 18–64 years) with Type 2 Diabetes Mellitus Medicaid National Database, 2022–2023.

		Depression		No Depression			
		N	%	N	%	Chi-Square	Prob
ALL		8561	11.5	66,199	88.5		
GLP1-RA Use						41.250	<0.001
	User	1936	12.9	13,017	87.1		
	Nonuser	6625	11.1	53,182	88.9		
Sex						427.089	<0.001
	Female	5768	13.5	36,822	86.5		
	Male	2793	8.7	29,377	91.3		
Age in Years						98.506	<0.001
	18–34 years	996	13.0	6643	87.0		
	35–44 years	1663	12.6	11,514	87.4		
	45–54 years	2548	12.1	18,459	87.9		
	55–64 years	3354	10.2	29,583	89.8		
Race and Ethnicity						514.256	<0.001
	NHW	4326	14.6	25,252	85.4		
	NHB	1785	9.9	16,316	90.1		
	Hispanic	936	10.3	8164	89.7		
	Other race	359	8.5	3857	91.5		
SDOH						74.461	<0.001
	Yes, problems	339	17.6	1583	82.4		
	No, problems	8222	11.3	64,616	88.7		
Capitation						2.276	0.131
	Capitated	2448	11.7	18,415	88.3		
	FFS	6113	11.3	47,784	88.7		
Plan Comprehensive						77.682	<0.001
	Plan Comp	6285	12.1	45,508	87.9		
	No Comp	2276	9.9	20,691	90.1		
Plan HMO						3.995	0.046
	Plan HMO	2357	11.8	17,554	88.2		
	No HMO	6204	11.3	48,645	88.7		
Plan PCCM						1.043	0.307
	Plan PCCM	527	11.9	3892	88.1		
	No PCCM	8034	11.4	62,307	88.6		
Obesity						101.445	<0.001
	Yes, obesity	4846	12.6	33,645	87.4		
	No, obesity	3715	10.2	32,554	89.8		
Alcohol Use						137.513	<0.001
	Alcohol user	526	18.3	2352	81.7		
	Nonuser	8035	11.2	63,847	88.8		
OUD						266.137	<0.001
	OUD	873	18.8	3760	81.2		
	No OUD	7688	11.0	62,439	89.0		
Tobacco Use						25.699	<0.001
	Tobacco user	110	17.9	503	82.1		
	Nonuser	8451	11.4	65,696	88.6		

Notes: Based on 74,760 adults aged 18–64 years, continuously enrolled in Medicaid with prescription drug coverage during 2022 and 2023, with Type 2 Diabetes Mellitus. Group differences in depression status were tested with chi-square statistic. Missing values include race and ethnicity and mental health coverage and are not presented in the table. Comp: Comprehensive; FFS: Fee-for-service; GLP1-RA: Glucagon-like Peptide-1 Receptor Agonists; HMO: Health maintenance organization; NHB: Non-Hispanic Black; NHW: Non-Hispanic White; OUD: Opioid use disorder; PCCM: Primary care case management; SDOH: Social determinants of health.

**Table 3:** Conditional, Marginal Estimates, and Doubly Robust Estimates of GLP-1 RA use from Logistic and Poisson Regressions on Newly Documented Depression Diagnosis Adults (18–64 years) with Type 2 Diabetes Mellitus, Medicaid National Database, 2022–2023.

Fully Adjusted Multivariable Logistic Regression on Newly Documented Depression Diagnosis (Conditional Association)			
**GLP1 Usage**	**aOR**	**95% CI**	**Prob**
GLP1			
Any GLP1-RA	1.10	[1.04, 1.16]	0.0013
No GLP1- RA Use (Ref)			
Marginal Association (Population-Average Effects) from Inverse Probability Weighted Logistic Regression			
GLP1	mOR	95% CI	Prob
Any GLP1-RA	1.09	[1.03, 1.15]	0.0049
No GLP1- RA Use (Ref)			
Doubly Robust Estimator from Inverse Probability Weighted Logistic Regression			
GLP1	DR-OR	95% CI	Prob
Any GLP1-RA	1.09	[1.03, 1.15]	0.0047
No GLP1- RA Use (Ref)			
Doubly Robust Estimator from Inverse Probability Weighted Poisson Regression			
GLP1	DR-RR	95% CI	Prob
Any GLP1-RA	1.08	[1.02, 1.13]	0.0041
No GLP1- RA Use (Ref)			

Notes: Based on 74,760 adults aged 18–64 years, continuously enrolled in Medicaid with prescription drug coverage during 2022 and 2023, with Type 2 Diabetes Mellitus. Abbreviations: GLP1-RA: Glucagon-like Peptide-1 Receptor Agonists; mOR: Marginal odds ratio representing the population-average effect and not covariate conditional; DR-OR: Double Robust Odds Ratio; DR-RR: Double Robust Risk Ratio.

## Data Availability

Merative MarketScan multistate Medicaid claims data must be purchased; therefore, the data is available upon request from the corresponding author.

## Supplementary Materials

The following supporting information can be downloaded at: https://www.mdpi.com/article/10.3390/healthcare14182971/s1. Figure S1. Study Cohort Selection Flow Diagram. Adults (age 18–64 years) with Type 2 Diabetes Mellitus, Medicaid National Database, 2022–2023; Figure S2. Study Timeline and Assessment Periods; Table S1. NDC Codes and ICD-10-CM Codes Used to Identify GLP-1 RA and Depression.

## Abbreviations

The following abbreviations are used in this manuscript:

CI	Confidence interval
GLP-1 RA	Glucagon-like peptide-1 receptor agonists
IPW	Inverse probability weighting
SD	Standard deviations
SMD	Standard mean difference
T2DM	Type 2 diabetes mellitus
OR	Odds ratio
