# Supplementary material for: Association Between Baseline Glucagon-like Peptide-1 Receptor Agonist Use and Newly Documented Depression Diagnosis in Adults with Type 2 Diabetes: A Multi-State Medicaid Cohort Study
Source: Healthcare (Basel). 2026 Sep 11;14(18):2971. doi: 10.3390/healthcare14182971 (PMC13606689; doi:10.3390/healthcare14182971)
Supplement: Supplementary file 1 [file healthcare-14-02971-s001.zip › healthcare-4490740-supplementary.pdf]

Supplementary Materials

Figure S1. Study Cohort Selection Flow Diagram

Adults (age 18-64 years) with Type 2 Diabetes Mellitus

Medicaid National Database, 2022-2023

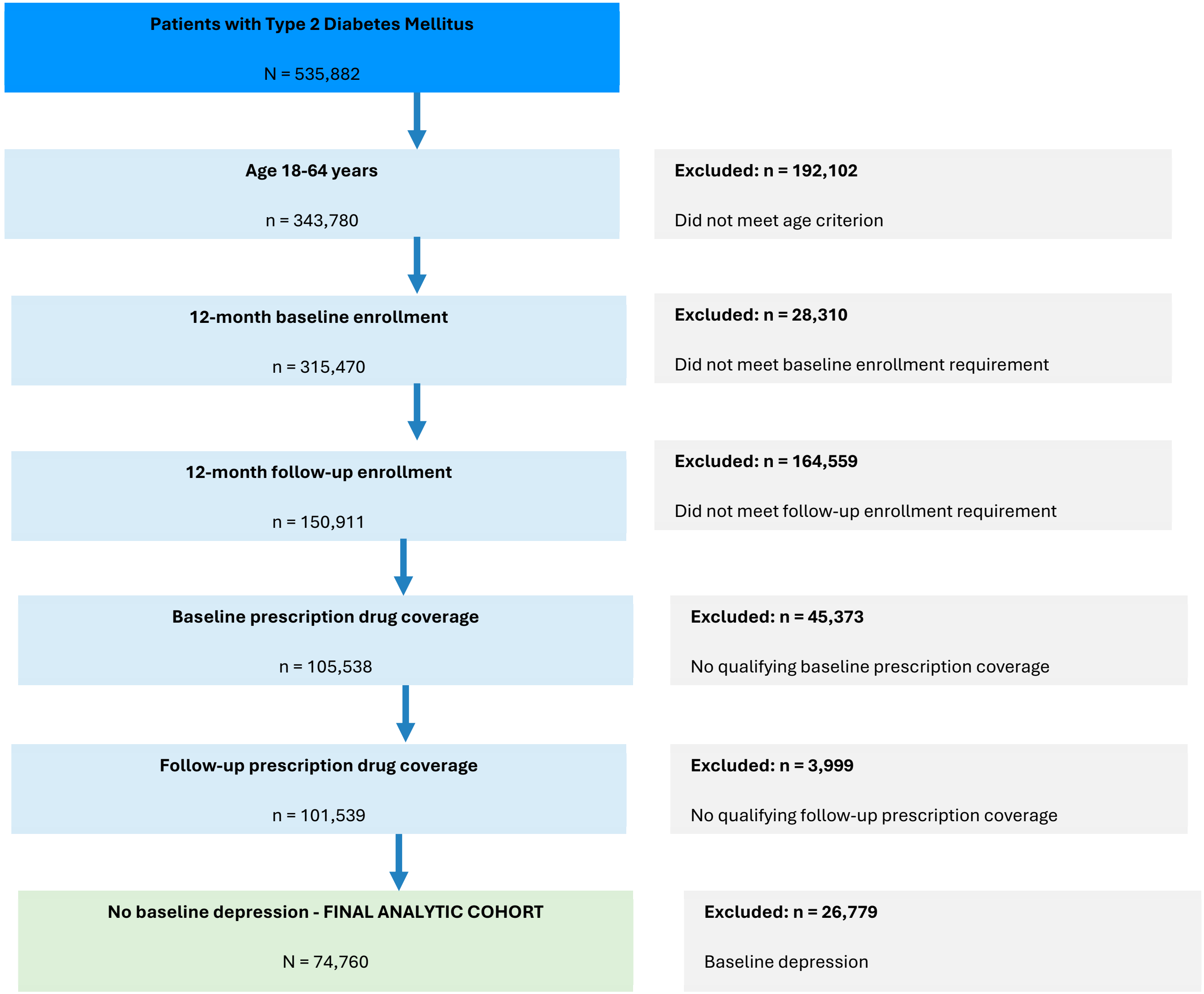

Figure S2. Study Timeline and Assessment Periods

Merative MarketScan Multi-State Medicaid Database, 2022–2023

BASELINE PERIOD<br>January 1–December 31, 2022		FOLLOW-UP PERIOD<br>January 1–December 31, 2023	
**Cohort Eligibility**<br>Adults aged 18–64 years with T2DM; continuous Medicaid enrollment with prescription drug coverage; no documented baseline depression.	**Exposure Assessment**<br>GLP-1 RA exposure assessed from outpatient pharmacy claims during 2022. At least one qualifying dispensing classified a beneficiary as exposed.	**Covariate Assessment**<br>Baseline demographic, insurance, social, behavioral, and clinical covariates assessed during the 2022 baseline period.	**Outcome Assessment**<br>Newly documented depression diagnosis identified from qualifying inpatient or outpatient ICD-10-CM claims during the 2023 follow-up period.

2022 baseline assessment → 2023 follow-up outcome ascertainment

Note. Exposure and baseline covariates were assessed during the 2022 baseline period. Outcome ascertainment occurred during the subsequent 2023 follow-up period. Continuous Medicaid enrollment with prescription drug coverage was required across both study years.

Abbreviations: GLP-1 RA, glucagon-like peptide-1 receptor agonist; ICD-10-CM, International Classification of Diseases, Tenth Revision, Clinical Modification; T2DM, type 2 diabetes mellitus.

Figure S2. Study timeline and assessment periods. GLP-1 RA exposure, cohort eligibility, and baseline covariates were assessed during the January 1 through December 31, 2022 baseline period. Newly documented depression diagnoses were assessed during the January 1 through December 31, 2023 follow-up period.

Supplementary Table S1. NDC Codes and ICD-10-CM Codes Used to Identify GLP-1 RA and Depression		
NDC codes to identify GLP-1 RA	Generic Name	NDC
	Albiglutide	00173086601, 00173086602, 00173086635, 00173086701, 00173086735
	Dulaglutide	00002143301, 00002143361, 00002143380, 00002143401, 00002143461, 00002143480, 00002223601, 00002223680, 00002318201, 00002318280, 50090348300, 50090348400, 50090546700, 50090645300, 50090645600, 50090657100
	Exenatide	00310651201, 00310652004, 00310652401, 00310653001, 00310653004, 00310654001, 00310654004, 54868538400, 54868538401, 66780021007, 66780021008, 66780021201, 66780021904, 66780022601, 68258894701, 68258894802
	Liraglutide	00169280015, 00169406012, 00169406013, 00169406090, 00169406099, 50090285300, 50090425700, 50090450300, 54569650700
	Lixisenatide	00024574702
	Semaglutide	00169413001, 00169413013, 00169413211, 00169413212, 00169413602, 00169418113, 00169430313, 00169430330, 00169430713, 00169430730, 00169431413, 00169431430, 00169450114, 00169450514, 00169451714, 00169452414, 00169452514, 00169477211, 00169477212, 00169477297, 50090582400, 50090594900, 50090605100
	Tirzepatide	00002145701, 00002145780, 00002146001, 00002146080, 00002147101, 00002147180, 00002148401, 00002148480, 00002149501, 00002149580, 00002150601, 00002150680
ICD-10-CM codes used to identify Depression	F320, F321, F322, F323, F324, F325, F328, F329, F330, F331, F332, F333, F3340, F3341, F3342, F338, F339	
